# PREVENT vs PCE thresholds for coronary artery calcium referral: Detection-optimized strategies in ELSA-Brasil

**DOI:** 10.1016/j.ajpc.2026.101646

**Published:** 2026-04-19

**Authors:** Daniel A. Added, Fernando Y. Cesena, Marcio H. Miname, Henrique Takematsu, Itamar de Souza Santos, Isabela M. Bensenor, Paulo A. Lotufo, Raul D. Santos

**Affiliations:** a Lipid Clinic, Heart Institute (InCor), University of São Paulo Medical School Hospital, São Paulo, SP, Brazil; b Center for Clinical and Epidemiological Research, University Hospital, University of São Paulo, 2565 Professor Lineu Prestes Avenue, São Paulo, SP 05508-000, Brazil; c Instituto Dante Pazzanese de Cardiologia, São Paulo, SP, Brazil

**Keywords:** Cardiovascular risk prediction, Risk stratification, Imaging-based prevention, Detection efficiency, Sex-based analysis, Preventive cardiology

## Abstract

**Background:**

Risk equations estimate 10-year ASCVD risk but were not designed to guide coronary artery calcium (CAC) testing, although guidelines consider CAC for risk refinement in borderline or intermediate-risk adults.

**Objectives:**

To compare the Predicting Risk of Cardiovascular Disease EVENTs (PREVENT) with Pooled Cohort Equations (PCE) for detecting CAC, identifying optimized CAC referral thresholds.

**Methods:**

In a cross-sectional analysis of 3477 ASCVD-free ELSA-Brasil participants, 10-year ASCVD risk was estimated using both tools, and reclassification assessed. Discrimination for CAC >0 and CAC ≥100 Agatston units was compared using area under receiver operating characteristic curve (AUC-ROC). Prespecified thresholds (PCE 7.5%; PREVENT 3.7% in men, 4.9% in women) and Youden-derived cutpoints were evaluated for sensitivity, specificity, scanning proportion, and number needed to scan (NNS).

**Results:**

Discrimination for CAC ≥100 was similar (AUC 0.81 vs 0.80 for PCE and PREVENT, respectively; p > 0.05). Using standard thresholds (7.5% for PCE; 3.7%/4.9% for PREVENT in men/women), 36.5% vs 38.8% of with CAC ≥100 were classified below cutpoints, more often in women (70.8% vs 67.4%). Youden-derived thresholds (5.6% vs 2.7%) increased sensitivity (73.3% vs 78.4%) but reduced specificity (74.1% vs 66.7%) and expanded referral eligibility (30.6% vs 38.2%), respectively.

**Conclusions:**

Both tools showed comparable discrimination for CAC ≥100, but standard cutpoints miss individuals, especially women. Lower, optimized thresholds (PCE 5.6%; PREVENT 2.7%) may improve detection but warrant further evaluation.

## 1 Introduction

Cardiovascular disease (CVD) remains the leading cause of death worldwide and a major contributor to global morbidity, underscoring the need for accurate risk stratification to guide the timely initiation and intensification of preventive interventions [1]. In contemporary practice, prevention often relies on complementary approaches: estimation of absolute risk using multivariable prediction models, and further risk refinement in selected individuals through assessment of subclinical atherosclerosis, most commonly with coronary artery calcium (CAC) imaging [2]. In contrast to multivariable risk equations that integrate numerous clinical and biochemical variables, CAC scoring provides a direct, imaging-based quantification of atherosclerotic burden.

To refine this clinical estimation, the recently introduced Predicting Risk of Cardiovascular Disease EVENTs (PREVENT) equations were designed to improve atherosclerotic cardiovascular disease (ASCVD) risk estimation by incorporating metabolic and renal biomarkers and removing race as a covariate, thereby addressing calibration drift and risk overestimation observed with the Pooled Cohort Equations (PCE).² Whether these design advances translate into meaningfully different decisions about lipid-lowering therapy or into more efficient selection of individuals for imaging-based risk refinement, however, remains uncertain. This gap is particularly significant given the prognostic capability of CAC, with CAC ≥100 Agatston units (AU) associated with 10-year ASCVD event rates that typically warrant statin therapy [[3], [4], [5]]. Accordingly, the 2019 ACC/AHA guideline considers CAC when treatment decisions are uncertain in adults at borderline or intermediate PCE-estimated risk [6]; the 2025 Brazilian Dyslipidemia Guideline adopts PREVENT as the preferred risk estimator and classifies individuals with CAC ≥100 AU as high risk irrespective of the absolute risk predicted by this tool.⁷ Nevertheless, CAC testing is typically considered after absolute risk stratification using event-based equations, and the thresholds currently applied to these tools have not been systematically validated for CAC referral [2,[6], [7], [8]].

These gaps are particularly relevant in Brazil, where access to imaging and competing health priorities differ from those of the U.S. cohorts from which PREVENT was derived. The Brazilian Longitudinal Study of Adult Health (ELSA-Brasil), a large cohort of apparently healthy civil servants at baseline, with standardized assessment of risk factors and CAC offers a suitable setting to evaluate how PREVENT and PCE perform as triage tools for CAC referral in a middle-income, multiethnic population broadly similar to adults typically considered for CAC screening [9,10].

Using data from ELSA-Brasil, we therefore sought to (1) compare PREVENT and PCE for detecting CAC ≥100 and CAC >0; (2) quantify risk reclassification across ACC/AHA-defined risk categories; and (3) identify empirically derived, efficiency-optimized thresholds that enhance detection of high-burden CAC while limiting unnecessary imaging, overall and by sex. We hypothesized that guideline-aligned thresholds for borderline-to-intermediate 10-year ASCVD risk, commonly used for CAC referral, miss a substantial proportion of individuals with CAC ≥100, particularly women, and that lower, data-driven cutoffs would yield more efficient and equitable CAC referral strategies in this Brazilian cohort.

## 2 Methods

### 2.1 Design and study population

**Fig. 1:**
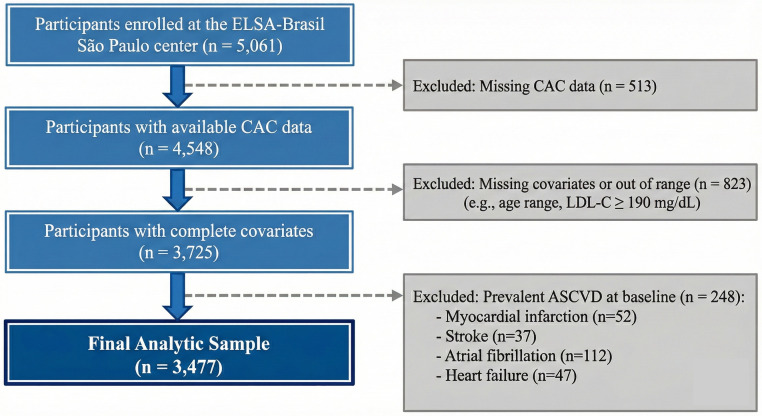
**Study Population and Analytical Flow**. This figure illustrates participant selection and analytical flow for the study. From the São Paulo center of ELSA-Brasil, ASCVD-free participants who underwent coronary artery calcium (CAC) scanning were included. Risk was estimated using the PCE and PREVENT equations, followed by evaluation of CAC presence and burden. The figure summarizes exclusions and the final analytic cohort used for comparisons of discrimination, classification, and CAC detection efficiency. Abbreviations: CAC = coronary artery calcium; ELSA-Brasil = Brazilian Longitudinal Study of Adult Health; LDL-C = low-density lipoprotein cholesterol; PCE = Pooled Cohort Equations; PREVENT = Predicting Risk of Cardiovascular Disease EVENTs.

We performed a cross-sectional analysis within the São Paulo center of ELSA-Brasil, a Brazilian cohort of civil servants and the only study site with coronary artery calcium (CAC) measurements. ELSA-Brasil is an ongoing cohort of 15,105 civil servants aged 35–74 years recruited between 2008 and 2010 in six Brazilian cities; the design and methods have been reported elsewhere [9]. For the present analysis, we included participants from the São Paulo center (n = 5061). We excluded individuals with missing CAC data (n = 513), missing mandatory covariates or values outside the prespecified input ranges required for PCE and PREVENT estimation (n = 823), and prevalent ASCVD at baseline (n = 248). The final analytic sample comprised 3477 participants (Fig. 1).

All participants provided written informed consent, and the institutional ethics committees approved the ELSA-Brasil study protocol.

### 2.2 Clinical and laboratory measurements

Sociodemographic, clinical, and laboratory variables were collected according to standardized ELSA-Brasil protocols [9]. Sociodemographic data included sex, age, education, and self-reported race/skin color (White, Mixed, Black, Asian and Indigenous). Smoking status was classified as never, past, or current. Body mass index (BMI) was analyzed continuously. Leisure-time physical activity was assessed using the long International Physical Activity Questionnaire (IPAQ) and classified according to World Health Organization (WHO) criteria as active (≥150 min/week of moderate-intensity or ≥75 min/week of vigorous-intensity, or equivalent), partly active, or inactive. All variables required for absolute ASCVD risk calculation were obtained prospectively. Blood pressure was measured three times at one-minute intervals using a validated oscillometric device (Omron HEM-705CP), and the mean of the final two readings was used for analysis. Hypertension was defined as systolic BP ≥140 mmHg or diastolic BP ≥90 mmHg, or current antihypertensive use. Diabetes mellitus was defined by self-reported diagnosis or glucose-lowering therapy, or laboratory criteria (fasting glucose ≥126 mg/dL, HbA1c ≥6.5%, or 2-h OGTT ≥200 mg/dL). Fasting glucose was measured by the hexokinase method; HbA1c by HPLC (Bio-Rad Variant II Turbo).

Venous samples were collected after a 12-hour fast. Total cholesterol, HDL-C, LDL-C, and triglycerides were quantified via enzymatic colorimetric assays (ADVIA 1200, Siemens). For PREVENT-specific metabolic and renal inputs, serum creatinine was measured by the Jaffe kinetic method and used to calculate eGFR using the 2021 CKD-EPI equation; albuminuria was indexed by the urinary albumin-to-creatinine ratio (UACR), measured in spot urine samples.

#### 2.2.1 CAC measurement

All participants underwent CAC scanning with a 64-slice multidetector CT system (Brilliance 64; Philips Healthcare, Best, the Netherlands). Following the scout acquisition, an ECG-gated prospective protocol was performed with a 120 kV tube voltage and tube current adjusted to body habitus. Images were reconstructed in 2.5-mm slices using standard filtered back-projection. CAC was quantified in Agatston units. The primary CAC outcome was high-burden CAC (CAC ≥100 AU). The secondary outcome was any detectable CAC (CAC >0).

### 2.3 Risk estimation

Ten-year ASCVD risk was calculated using the PCE (2013) and PREVENT (2024) equations. In ELSA-Brasil, systematic measurement of plasma lipids, HbA1c, and UACR enabled the full implementation of PREVENT metabolic-renal components [2]. The U.S. Social Deprivation Index was not applied due to a lack of validation in Brazil. Risk estimates were categorized according to guideline strata (<5%, 5.0–7.4%, 7.5–19.9%, ≥20%) [6], the conventional PCE treatment threshold of 7.5% [6], and PREVENT-specific cutoffs statistically proposed to approximate PCE 7.5% (≥3.7% for men and ≥4.9% for women) [2]. We also evaluated empirically derived, detection-oriented thresholds obtained from Youden’s index for each equation, both in the overall population and separately in men and women. To examine risk stratification and reclassification, we cross-tabulated PREVENT and PCE across guideline-defined risk strata and calculated agreement, as well as upward and downward reclassification proportions, overall and within CAC categories (CAC =0, CAC 1–99, CAC ≥100).

### 2.4 Statistical analysis

Baseline characteristics were compared between men and women using Wilcoxon rank-sum tests for continuous variables and χ² tests for categorical variables. Normality was assessed using Shapiro–Wilk tests. Discrimination for CAC ≥100 and CAC >0 was assessed using the area under the receiver operating characteristic curve (AUC-ROC), with comparisons between PREVENT and PCE performed using DeLong’s test for correlated ROC curves.

Diagnostic performance at pre-specified and empirically derived thresholds was summarized by sensitivity, specificity, positive predictive value (PPV), negative predictive value (NPV), proportion of the cohort eligible for scanning (proportion above the cutoff), and number needed to screen (NNS). NNS was calculated as the ratio of the number of individuals above a given threshold to the number of participants with the relevant CAC outcome (CAC ≥100 or CAC >0) above that threshold. For each alternative strategy, we quantified the incremental efficiency cost as the number of extra scans per additional CAC ≥100 detected, defined as the difference in the number of individuals above the reference threshold divided by the difference in the number of detected CAC ≥100 cases relative to the reference threshold.

Youden cut points and 95% confidence intervals (CIs) were obtained using 1000 bootstrap resamples. Prespecified sex-stratified analyses were conducted. AUCs were estimated separately in men and women, and between-sex differences in discrimination were evaluated using DeLong’s test for independent samples. Paired proportions were compared using McNemar’s test. All analyses were performed in R version 4.3.2 (R Foundation, Vienna). A two-sided p-value <0.05 was considered statistically significant.

## 3 Results

### 3.1 Baseline characteristics

Table 1 summarizes the characteristics of the 3477 participants (median age 51 years; 54.7% women). Men had higher systolic blood pressure and a greater prevalence of current smoking and diabetes, whereas women had higher HDL cholesterol levels and a higher UACR. Most participants had no detectable CAC, particularly women. High-burden CAC (CAC ≥100) was more frequently observed in men. Ten-year ASCVD risk estimates derived from both PREVENT and PCE were higher in men than in women. Baseline characteristics of included and excluded participants are compared in **Supplementary Table 1.**

**Table 1:** Baseline Characteristics of the Study Population (N = 3477).

	Total (n = 3477)	Men (n = 1574)	Women (n = 1903)
**Demographics**			
Age (years)	51.0 [45.5–56.5]	50.0 [46.0–57.0]	51.0 [46.0–57.0]
White	2058 (59.2%)	904(57.5%)	1154(60.9%)
College graduate or higher	1526 (43.9%)	592 (37,6%)	934 (49.1%)
**Clinical Characteristics**			
Systolic Blood Pressure (mmHg) *	117.5 [107.0–127.5]	122.5 [113.0–133.4]	113.0 [104.8–124.0]
Body Mass Index (kg/m²)	26.7 [24.1–29.8]	26.8 [24.3–29.4]	26.6 [23.9–30.1]
Physically Active	1718 (49.4%)	787(50.1%)	931 (48.9%)
Hypertension	833 (23.9%)	385 (24.5%)	448 (23.5%)
Diabetes *	523 (15.0%)	281 (17.9%)	242 (12.7%)
Current Smoking *	565 (16.2%)	267 (17.0%)	298 (15.6%)
Statin Use	431 (12.4%)	186 (11.8%)	245 (12.9%)
**Laboratory Data**			
Total Cholesterol (mg/dL)	199 [176.0–223.0]	198 [175.0–223.0]	199 [177.0–223.0]
LDL-C (mg/dL)	118 [98.0–140.0]	120 [99.0–141.0]	117 [97.0–139.0]
HDL-C (mg/dL) *	51 [44.0–60.0]	47 [41.0–54.0]	56 [47.0–65.0]
Triglycerides (mg/dL) *	112 [81.0–157.0]	125 [87.0–175.0]	103 [75.0–145.0]
eGFR (mL/min/1.73 m²)	74.2 [62.4–88.9]	74.2 [61.5–88.9]	74.7 [62.8–89.5]
UACR (mg/g)*	6.6 [5.1–8.5]	5.1 [4.3–6.3]	7.6 [6.5–9.2]
Hemoglobin A1c (%)	5.2 [4.9–5.6]	5.2 [4.9–5.6]	5.2 [4.9–5.6]
**ASCVD Risk Scores**			
PREVENT 10-Year Risk (%)*	**2.0 [0.8–4.7]**	**2.7 [1.5–4.6]**	**1.6 [0.9–3.0]**
PCE 10-Year Risk (%)*	**2.9 [1.2–6.9]**	**5.5 [2.7–10.5]**	**1.6 [0.7–3.6]**
**CAC**			
CAC = 0 *	2428 (69.8%)	908 (57.7%)	1520 (79.9%)
CAC 1–99 *	701 (10.2%)	407 (25.8%)	294 (15.5%)
CAC ≥ 100 *	348 (10.0%)	259 (16.5%)	89 (4.6%)

Baseline demographic and clinical characteristics of ASCVD-free participants from the São Paulo center of ELSA-Brasil, stratified by sex. Variables include cardiovascular risk factors, laboratory measures, and comorbidities. Continuous variables are presented as median (interquartile range) and categorical variables as counts and percentages. Comparisons between men and women were performed for all variables; statistically significant results (p < 0.05) are indicated by the (*) symbol.

**Abbreviations:** CAC = coronary artery calcium; eGFR = estimated glomerular filtration rate; Hemoglobin A1c = glycated hemoglobin; HDL-C = high-density lipoprotein cholesterol; LDL-C = low-density lipoprotein cholesterol; PCE = Pooled Cohort Equations; PREVENT = Predicting Risk of Cardiovascular Disease EVENTs; UACR = urinary albumin-to-creatinine ratio.

### 3.2 Model discrimination

**Fig. 2:**
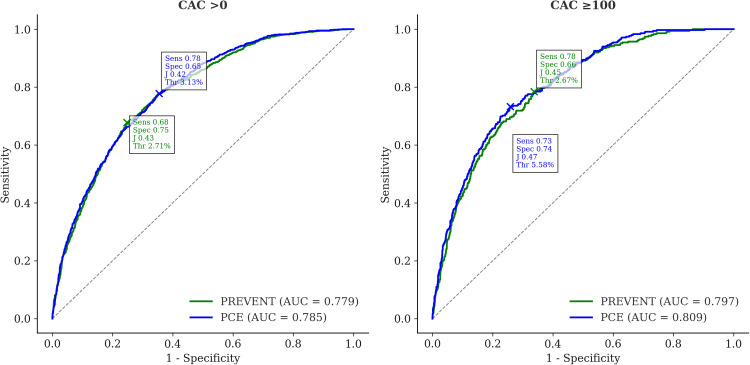
**Discrimination for Coronary Artery Calcium**. Receiver-operating characteristic curves compare the discrimination of PREVENT and PCE for detecting CAC >0 and CAC ≥100. Both models demonstrate similar area under the curve values, indicating comparable discrimination. Abbreviations: AUC = area under the curve; CAC = coronary artery calcium; J = Youden index; PCE = Pooled Cohort Equations; PREVENT = Predicting Risk of Cardiovascular Disease EVENTs; ROC = receiver-operating characteristic; Sens = sensitivity; Spec = specificity; Thr = threshold.

Fig. 2 shows similar discrimination between PREVENT and PCE for CAC >0 (AUC 0.78 vs 0.79; p = 0.800) and for CAC ≥100 (AUC 0.800 vs 0.810; p = 0.490). CAC >0 discrimination differed by sex, with AUC 0.73 in men and 0.80 in women for PREVENT (p = 0.001) and 0.73 vs 0.79 for PCE (p = 0.005). For CAC ≥100, no sex differences were observed for either equation (both comparisons p > 0.90).

### 3.3 Risk categorization and reclassification

Agreement between PREVENT and PCE across guideline-based risk categories (<5%, 5.0–7.4%, 7.5–19.9%, ≥20%) was 70.0% (**Supplementary Table 2**). Among discordant classifications (30.0%), PREVENT reassigned 29.3% of participants to a lower-risk category and 0.9% to a higher-risk category, with most downward transitions occurring from ≥7.5% to <7.5% (24.6%).

**Fig. 3:**
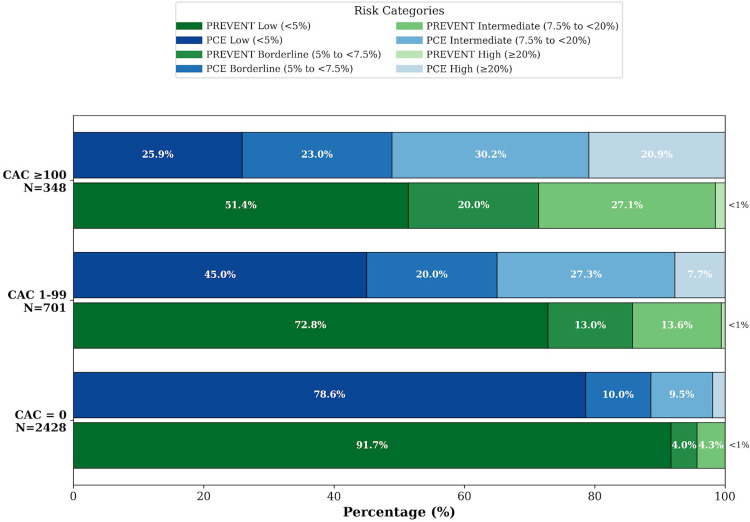
**Distribution of Estimated 10-Year ASCVD Risk Across CAC Strata**. This figure shows the distribution of 10-year ASCVD risk categories estimated by PREVENT and PCE across CAC strata (CAC = 0, CAC >0, and CAC ≥100 Agatston units). Risk categories were defined as low (<5%), borderline (5% to <7.5%), intermediate (7.5% to <20%), and high (≥20%), consistent with contemporary guideline-based thresholds. Individuals with CAC ≥100 frequently fall within low or borderline estimated risk categories, despite the presence of substantial subclinical atherosclerosis. Abbreviations: ASCVD = atherosclerotic cardiovascular disease; CAC = coronary artery calcium; PCE = Pooled Cohort Equations; PREVENT = Predicting Risk of Cardiovascular Disease EVENTs.

Among participants with CAC ≥100, 51.4% were classified as <5% risk by PREVENT compared with 25.9% by PCE (Fig. 3).

### 3.4 Classification of CAC ≥100 relative to referral thresholds

**Fig. 4:**
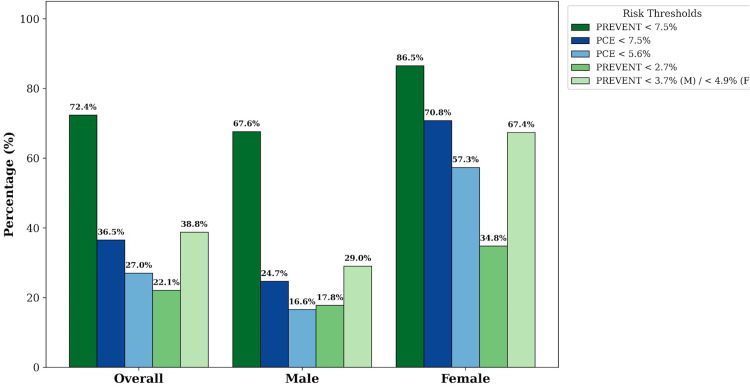
**Proportion of Patients With CAC ≥100 Classified Below Risk Thresholds**. Bar graph showing the percentage of patients with CAC ≥100 categorized below predefined PREVENT and PCE risk thresholds in the overall cohort and stratified by sex. PREVENT <7.5% identified 72.4% overall (67.6% men, 86.5% women). PCE <7.5% identified 36.5% overall (24.7% men, 70.8% women), and PCE <5.6% identified 27.0% overall (16.6% men, 57.3% women). PREVENT <2.7% identified 22.1% overall (17.8% men, 34.8% women). Sex-specific PREVENT thresholds (<3.7% men; <4.9% women) identified 38.8% overall (29.0% men, 67.4% women). Abbreviations: CAC = coronary artery calcium; PCE = Pooled Cohort Equations; PREVENT = Predicting Risk of Cardiovascular Disease EVENTs.

Using the conventional PCE 7.5% threshold and the AHA-recommended sex-specific PREVENT thresholds (3.7% in men; 4.9% in women), 36.5% and 38.8% of individuals with CAC ≥100 were classified below the respective cutpoints (p = 0.186 by McNemar’s test) (Fig. 4).

Among women, 70.8% were below the PCE 7.5% threshold and 67.4% were below the sex-specific PREVENT threshold (p = 0.510).

Across evaluated decision thresholds, the proportion of individuals with CAC ≥100 classified below threshold ranged from 22.1% to 72.4% (Figure 4 **and Supplementary Table 3**).

Specifically, 72.4% were below PREVENT <7.5%, 36.5% were below PCE <7.5%, 27.0% were below the Youden-derived PCE threshold (5.6%), and 22.1% were below the Youden-derived sex-neutral PREVENT threshold (2.7%).

### 3.5 Threshold performance for CAC ≥100

Table 2 summarizes the diagnostic and operational performance of PCE and PREVENT thresholds for identifying CAC ≥100, assuming that individuals classified above each threshold would be referred for CAC testing.

**Table 2:** Performance Metrics of PREVENT and PCE for CAC ≥100 Across Thresholds.

Population	Model/Threshold	Sens % (95% CI)	Spec % (95% CI)	PPV %	NPV %	NNS	CAC ≥100 n (%)	Extra Scans per Additional CAC ≥100 Detectionᵃ	Pop. Screened %
**Both sexes**	**PCE Intermediate (7.5 Ref)**	63.5 (58.2–68.6)	81.8 (80.4–83.1)	27.9	95.3	3.6	221 (63.5)	Ref	22.8
	PCE Borderline (5.0)	74.1 (69.2–78.7)	71.1 (69.5–72.7)	22.2	96.1	4.5	258 (74.1)	10.0	33.4
	PCE Youden (5.6)	73.3 (68.3–77.9)	74.1 (72.5–75.6)	23.9	96.1	4.2	255 (73.3)	8.0	30.6
	PREVENT Youden Both (2.7)	78.4 (74.1–82.8)	66.7 (65.0–68.3)	20.6	96.4	4.9	273 (78.4)	10.3	38.2
**Men**	**PREVENT (3.7 - Ref)**	71.0 (65.5–76.6)	71.6 (69.2–74.1)	33.0	92.6	3.0	184 (71.0)	Ref	35.4
	PREVENT Youden Both (2.7)	82.6 (78.0–87.2)	56.7 (54.0–59.3)	27.3	94.3	3.7	214 (82.6)	7.5	49.6
	PREVENT Youden Male (4.1)	67.6 (61.9–73.3)	76.1 (73.8–78.4)	35.8	92.3	2.8	175 (67.6)	6.8	31.1
**Women**	**PREVENT(4.9 - Ref)**	32.6 (22.8–42.3)	89.5 (88.1–90.9)	13.2	96.4	7.6	29 (32.6)	Ref	11.5
	PREVENT Youden Both (2.7)	66.3 (56.5–76.1)	72.8 (70.7–74.8)	10.7	97.8	9.4	59 (66.3)	10.9	28.7
	PREVENT Youden Female (1.6)	93.3 (88.0–98.5)	53.3 (51.0–55.5)	8.9	99.4	11.2	83 (93.3)	13.1	48.9

Diagnostic and operational performance of guideline-aligned and detection-optimized PREVENT and PCE thresholds for identifying CAC ≥100 Agatston units. Metrics include sensitivity, specificity, positive and negative predictive values, proportion screened (reported overall and within sex strata), number needed to scan (NNS), and the number and proportion of CAC ≥100 cases above each threshold. These measures quantify trade-offs between detection yield and imaging burden across prespecified and Youden-derived cutpoints.

ᵃExtra scans per additional CAC ≥100 detected denote the incremental number of CAC examinations required to identify one additional CAC ≥100 case relative to the reference threshold within each analytic stratum.

**Abbreviations:** CAC = coronary artery calcium; CI = confidence interval; NNS = number needed to scan; NPV = negative predictive value; PCE = Pooled Cohort Equations; Pop. = population; PPV = positive predictive value; PREVENT = Predicting Risk of Cardiovascular Disease EVENTs; Sens = sensitivity; Spec = specificity.

Using the conventional PCE 7.5% threshold, 22.8% of all participants would be referred for CAC scanning, with a sensitivity of 63.5%, a specificity of 81.8%, and a NNS 3.6 for detecting CAC ≥100.

Lowering the PCE threshold to 5.0% increased sensitivity to 74.1% and expanded referral to 33.4% of the cohort, with reduced specificity (71.1%) and an incremental efficiency cost of 10 additional scans per extra CAC ≥100 detected relative to the 7.5% threshold.

The Youden-derived PCE threshold (5.6%) yielded a sensitivity of 73.3%, a specificity of 74.1%, a referral rate of 30.6% of participants, and an incremental cost of 8 additional scans per extra CAC ≥100 detection.

The sex-neutral PREVENT Youden threshold (2.7%), applied to both sexes, resulted in a sensitivity of 78.4%, a specificity of 66.7%, a referral of 38.2% of participants, and a NNS of 4.9, with 10.3 additional scans per extra CAC ≥100 detected relative to PCE 7.5%.

### 3.6 Sex-Stratified prevent thresholds

Among men, the guideline-aligned PREVENT threshold of 3.7% yielded sensitivity of 71.0%, specificity of 71.6%, referral of 35.4% of men, and NNS of 3.0.

Applying the sex-neutral 2.7% threshold to men increased sensitivity to 82.6% but reduced specificity to 56.7%, with 49.6% of men above threshold and 7.5 additional scans required per extra CAC ≥100 detected (NNS 3.7).

The male-specific Youden threshold of 4.1% produced sensitivity of 67.6%, specificity of 76.1%, referral of 31.1% of men, and NNS of 2.8.

Among women, the guideline-aligned PREVENT threshold of 4.9% resulted in sensitivity of 32.6%, specificity of 89.5%, referral of 11.5% of women, and NNS of 7.6.

Applying the sex-neutral 2.7% threshold to women increased sensitivity to 66.3% and reduced specificity to 72.8%, with 28.7% of women above threshold and 10.9 additional scans required per extra CAC ≥100 detection with a NNS 9.4.

The female-specific Youden threshold of 1.6% further increased sensitivity to 93.3% but reduced specificity to 53.3%, expanding eligibility to 48.9% of women and requiring 8.6 additional scans per extra detection (NNS 11.2).

### 3.7 Secondary outcome: CAC >0

Results for CAC >0 paralleled those observed for CAC ≥100 (**Supplementary Tables 4 and 5**). Using the conventional PCE 7.5% threshold, 46.3% of individuals with CAC >0 were classified above referral threshold, compared with 17.7% using sex-specific PREVENT thresholds (p < 0.001), with overall concordance of 71.0%.

Sensitivity and specificity for detecting CAC >0 were 55.0% and 79.0% in men and 29.5% and 93.0% in women with PREVENT (3.7% men; 4.9% women). Using PCE 7.5%, overall sensitivity and specificity were 46.3% and 87.4%, respectively.

NNS was similar across models at recommended thresholds (1.5 in men and 1.9 in women).

## 4 Discussion

In this cross-sectional analysis of CAC-measured civil servants from the São Paulo center of ELSA-Brasil, PREVENT and PCE showed similar discrimination for identifying individuals with CAC ≥100. However, threshold selection, rather than equation choice, was the primary factor influencing CAC detection, imaging eligibility, and operational efficiency. This distinction is clinically relevant because PREVENT and PCE were developed to estimate future ASCVD events, not to guide selection for CAC testing. Our findings should therefore be interpreted as addressing a clinical implementation question within this cohort: how treatment-based and detection-optimized thresholds perform when used to guide CAC referral.

PREVENT more frequently classified participants, including those with CAC ≥100, into lower guideline-based risk strata than PCE. This downward redistribution is expected and reflects the recalibration framework of PREVENT rather than a novel observation [2]. The clinically relevant issue, therefore, is not whether PREVENT yields lower estimated risk overall, but how clinically meaningful subclinical atherosclerosis is distributed across the risk strata defined by each equation. In our data, despite generating lower estimated risk, PREVENT did not result in greater enrichment of individuals with substantial CAC in higher-risk categories, and a considerable proportion of participants with CAC ≥100 remained classified within lower-risk strata.

This is clinically relevant because CAC is a direct measure of atherosclerotic burden with established prognostic significance. Prior cohort studies have shown a strong and independent association between increasing CAC burden and subsequent ASCVD events, with CAC ≥100 consistently identifying individuals with substantially higher event rates [3,5]. In MESA, CAC ≥100 corresponded to a 10-year ASCVD risk above the conventional treatment threshold of 7.5% [5,6]. Thus, better calibration of an event-based equation does not diminish the clinical value of CAC; rather, it highlights the need to determine whether currently used risk thresholds identify those most likely to harbor high-burden CAC. The prospective design and standardized phenotyping of ELSA-Brasil support interpretation of these comparisons within a rigorously characterized cohort [[9], [10], [11]].

A central implication of this study is that treatment-oriented event thresholds should not be assumed to be optimal imaging-referral thresholds. The widely adopted 7.5% 10-year ASCVD risk threshold for PCE became a guideline anchor because it approximated a level of observed event risk at which statin therapy showed favorable net clinical benefit and cost-effectiveness, not because it optimized detection of structural atherosclerosis [6,[12], [13], [14], [15]]. Accordingly, the threshold best suited to identifying clinically relevant CAC need not coincide with the threshold at which pharmacologic therapy becomes cost-effective, a distinction that may become increasingly important as PREVENT is incorporated into contemporary prevention guidelines without explicit CAC referral criteria [7,8].

The Youden-derived optimal PCE threshold for detecting CAC ≥100 (5.6%) emerged independently of guideline categories yet was numerically close to the lower boundary of the borderline risk range (5%). Prior studies have shown that CAC provides substantial net reclassification benefit among individuals in borderline and intermediate risk categories [2,9]. Our findings extend this concept by suggesting that, from a detection-oriented perspective, the most informative PCE threshold for CAC referral may lie near the borderline boundary rather than the intermediate threshold traditionally used to guide statin therapy. In parallel, for PREVENT, a lower sex-neutral threshold (2.7%) provided a pragmatic balance between sensitivity, specificity, and referral burden across both sexes, suggesting that it may represent a candidate unified gate for CAC testing in this population when using this tool.

In CAUGHT-CAD, a CAC-guided strategy in intermediate-risk patients improved LDL-C control and attenuated plaque progression compared with usual care, but did not derive or compare PCE- or PREVENT-based selection thresholds.¹⁶ Our analysis addresses this earlier step in the care pathway by evaluating how threshold choice shapes CAC detection and operational efficiency before imaging is performed. Whether broader CAC testing based on such thresholds improves outcomes, cost-effectiveness, or net clinical benefit remains unknown and will require prospective validation and formal economic modeling [15,16].

A particularly important finding was the sex-specific effect of threshold choice. At guideline-aligned thresholds, sensitivity for detecting CAC ≥100 was substantially lower in women than in men. Lowering thresholds improved detection in women but increased referral volume and reduced specificity. Importantly, this pattern should not be interpreted as evidence of inferior discrimination for CAC ≥100 in women, because discrimination at that threshold did not significantly differ by sex. Instead, the observed difference in detection is more consistent with threshold-position effects than with demonstrable sex differences in overall model discrimination, although limited statistical power for CAC ≥100 in women should be acknowledged [[10], [11], [17]].

From an implementation perspective, these findings support evaluation of a unified, sex-neutral PREVENT gate. While sex-specific thresholds may increase sensitivity in women, they also produce markedly different referral proportions between sexes, potentially complicating workflow and scanner allocation. In our cohort, a unified PREVENT threshold of 2.7% represented a pragmatic operational cutpoint with measurable trade-offs in both sexes. More broadly, referral volume varied nearly threefold across evaluated thresholds, and even modest downward shifts in referral gates substantially increased imaging eligibility. In resource-constrained settings, such changes may meaningfully affect scanner capacity, workflow logistics, and cost. By incorporating referral proportion, extra scans per additional CAC ≥100 detected, and number needed to scan, our analysis frames CAC referral not only as a discrimination problem, but also as a practical implementation decision.

External data further contextualize these findings. PREVENT has been shown to shift risk categorization toward lower strata while high-risk CAC remains prevalent among individuals labeled short-term “low risk [18].” Thus, the clinically relevant issue is not simply the absolute level of estimated risk, but how structural atherosclerosis is distributed across the categories used to guide preventive action. Importantly, our findings should not be interpreted as supporting lower treatment thresholds when PREVENT is used. Treatment thresholds should remain anchored to prospective evidence of clinical benefit and cost-effectiveness. Rather, our results suggest that substantial subclinical disease may remain under-recognized within PREVENT-defined lower-risk categories, reinforcing the need to distinguish between thresholds used to initiate therapy and thresholds used to select patients for imaging. In MESA, even within the PREVENT <5% category, participants with baseline CAC >0 experienced higher ASCVD incidence than those with CAC =0 within the same risk stratum [19]. Similar observations, together with prior ELSA-Brasil analyses demonstrating CAC among favorable risk profiles, support empiric evaluation of threshold positioning and sex-stratified referral strategies [11].

This study has limitations. First, the analysis was restricted to the São Paulo center of ELSA-Brasil and to a cohort of civil servants, creating a specific socioeconomic and occupational sampling frame that limits inference to this CAC-measured Brazilian cohort rather than to the broader Brazilian population. Second, substantial exclusions were required because of missing CAC data, prevalent ASCVD, and missing or out-of-range inputs required for PCE or PREVENT estimation; although necessary for valid score calculation, these exclusions may affect generalizability. Third, PREVENT was not implemented in its full form because the social deprivation component was not applied owing to lack of validation in Brazil; however, analyses from the original PREVENT derivation study suggest that inclusion of this component results in relatively modest changes in risk estimation and model performance.² Fourth, the cross-sectional design and absence of adjudicated ASCVD outcomes preclude evaluation of event-based calibration and prevent any inference that detection-optimized thresholds improve downstream clinical events, cost-effectiveness, or net clinical benefit. Finally, we did not quantify downstream treatment changes, radiation exposure, adverse effects, or health-system costs under alternative referral strategies. Nonetheless, standardized phenotyping, rigorous CAC assessment, and prespecified operational performance metrics support the internal validity of the observed trade-offs and underscore the need for prospective validation, replication, and health-economic modeling [10].

In this CAC-measured Brazilian cohort, PREVENT and PCE showed similar discrimination for identifying CAC ≥100, but currently used risk thresholds did not identify a substantial proportion of individuals with clinically relevant subclinical atherosclerosis, particularly women. These findings suggest that treatment-oriented risk cutoffs may not be optimal for CAC referral and support evaluation of lower, detection-oriented referral thresholds to better align imaging strategies with underlying atherosclerotic burden.

## Abbreviation LIST

ASCVD = atherosclerotic cardiovascular disease

AUC-ROC = area under the receiver operating characteristic curve

AU = Agatston units

BMI = body mass index

CAC = coronary artery calcium

CI = confidence interval

CT = computed tomography

CVD = cardiovascular disease eGFR = estimated glomerular filtration rate

HbA1c = glycated hemoglobin

HDL-C = high-density lipoprotein cholesterol

IPAQ = International Physical Activity Questionnaire

LDL-C = low-density lipoprotein cholesterol

NNS = number needed to scan

NPV = negative predictive value

PCE = Pooled Cohort Equations

PPV = positive predictive value

PREVENT = Predicting Risk of Cardiovascular Disease EVENTs

UACR = urinary albumin-to-creatinine ratio

**Figure:**
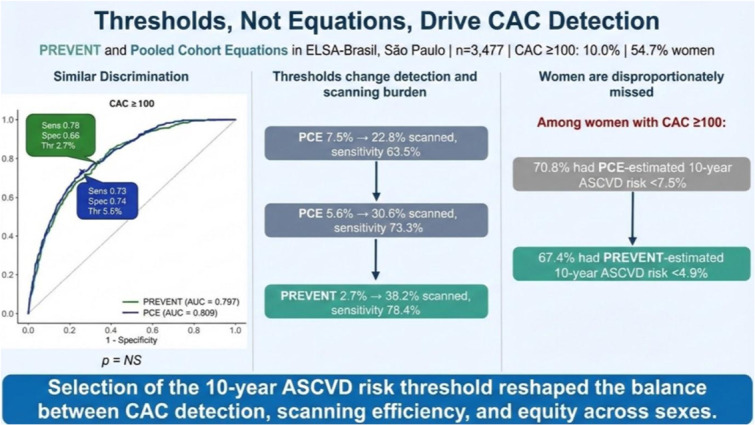
**Central Illustration. Thresholds, Not Equations, Drive CAC Detection**.In this cross-sectional analysis of 3477 ASCVD-free participants from the São Paulo center of ELSA-Brasil, PREVENT and PCE showed similar discrimination for identifying high-burden coronary artery calcium (CAC ≥100 AU), but the 10-year ASCVD risk threshold selected for CAC referral substantially changed detection yield and scanning demand. Using the conventional PCE threshold of 7.5%, 22.8% of participants would be referred for CAC testing, with sensitivity of 63.5% for CAC ≥100. Lowering the threshold to the Youden-derived PCE cutoff of 5.6% increased sensitivity to 73.3% and referral to 30.6%, whereas a sex-neutral PREVENT threshold of 2.7% increased sensitivity to 78.4% and referral to 38.2%. Standard thresholds also missed a substantial proportion of participants with CAC ≥100, particularly women: 70.8% of women with CAC ≥100 had a PCE-estimated 10-year ASCVD risk <7.5%, and 67.4% had a PREVENT-estimated 10-year ASCVD risk <4.9%. Overall, selection of the 10-year ASCVD risk threshold reshaped the balance between CAC detection, scanning efficiency, and equity across sexes. Abbreviations: ASCVD = atherosclerotic cardiovascular disease; AUC = area under the curve; CAC = coronary artery calcium; PCE = Pooled Cohort Equations; PREVENT = Predicting Risk of Cardiovascular Disease EVENTs.

## Funding

This work used data from the Brazilian Longitudinal Study of Adult Health (ELSA-Brasil), funded by the Brazilian National Council for Scientific and Technological Development (CNPq) and the Brazilian Ministry of Health, Brasília, DF, Brazil. Relevant grants for the São Paulo center and study waves used include 01 06 0115-00 and 01 10 0773-00. Productivity research scholarships from CNPq were awarded to Isabela M. Bensenor (307719/2006-5), Itamar de Souza Santos (306615/2022-3), Raul D. Santos Filho (303771/2023-2), and Paulo A. Lotufo (305822/2016-0).

## Ethical approval

This study reports data from human participants enrolled in the Brazilian Longitudinal Study of Adult Health (ELSA-Brasil). The study protocol was approved by the institutional ethics committees of all participating centers, including the Research Ethics Committee of the University of São Paulo. All participants provided written informed consent. The present analysis was conducted in accordance with these approvals and with the principles of the Declaration of Helsinki.

## Author agreement

The undersigned authors certify that:

Originality and Exclusive Submission

The manuscript submitted is an original work and has not been previously published, in whole or in part, except as an abstract or preprint where permitted. The manuscript is not currently under consideration for publication elsewhere.

## Ethical standards

The research reported in this manuscript was conducted in accordance with internationally accepted ethical standards. Where applicable, approval was obtained from the appropriate institutional review boards or ethics committees, and all participants provided informed consent.

## Copyright and publication rights

Upon acceptance of the manuscript for publication, the authors agree to comply with the journal's copyright policies as required by the publisher (Elsevier), including transfer or licensing of publication rights as specified by the journal.

## CRediT authorship contribution statement

**Daniel A. Added:** Writing – original draft, Methodology, Formal analysis, Data curation, Conceptualization. **Fernando Y. Cesena:** Writing – review & editing, Visualization, Supervision. **Marcio H. Miname:** Writing – review & editing, Visualization, Supervision. **Henrique Takematsu:** Writing – review & editing. **Itamar de Souza Santos:** Writing – review & editing, Visualization, Supervision. **Isabela M. Bensenor:** Writing – review & editing, Visualization, Supervision. **Paulo A. Lotufo:** Writing – review & editing, Visualization, Supervision. **Raul D. Santos:** Writing – review & editing, Visualization, Supervision.

## Declaration of competing interest

The authors declare the following financial interests/personal relationships which may be considered as potential competing interests:

Itamar de Souza Santos reports financial support was provided by National Council for Scientific and Technological Development. Isabela M. Bensenor reports financial support was provided by National Council for Scientific and Technological Development. Paulo A. Lotufo reports was provided by National Council for Scientific and Technological Development. Raul D. Santos reports was provided by National Council for Scientific and Technological Development. Raul D. Santos reports a relationship with Amgen Inc that includes: consulting or advisory, non-financial support, and speaking and lecture fees. Raul D. Santos reports a relationship with Ache Pharmaceutical Laboratories SA that includes: board membership, funding grants, and non-financial support. Raul D. Santos reports a relationship with Arrowhead Pharmaceuticals Inc that includes: funding grants and non-financial support. Raul D. Santos reports a relationship with Biolab Pharmaceuticals that includes: funding grants and non-financial support. Raul D. Santos reports a relationship with Daiichi Sankyo Inc that includes: funding grants and non-financial support. Raul D. Santos reports a relationship with Esperion Therapeutics Inc that includes:. Raul D. Santos reports a relationship with Eli Lilly and Company that includes: funding grants and non-financial support. Raul D. Santos reports a relationship with Ionis Pharmaceuticals Inc that includes: funding grants and non-financial support. Raul D. Santos reports a relationship with Merck & Co Inc that includes: funding grants and non-financial support. Raul D. Santos reports a relationship with Novo Nordisk Inc that includes: funding grants and non-financial support. Raul D. Santos reports a relationship with Novartis that includes: funding grants and non-financial support. Raul D. Santos reports a relationship with Torrent Pharmaceuticals Limited that includes: funding grants and non-financial support. Raul D. Santos reports a relationship with Sanofi that includes: funding grants and non-financial support. Raul D. Santos reports a relationship with Ultragenyx Pharmaceutical Inc that includes: funding grants and non-financial support. The authors declare no other relationships or activities that could appear to have influenced the submitted work. If there are other authors, they declare that they have no known competing financial interests or personal relationships that could have appeared to influence the work reported in this paper.

## Appendix Supplementary materials

Image, application 1
