# Supplementary material for: PREVENT vs PCE thresholds for coronary artery calcium referral: Detection-optimized strategies in ELSA-Brasil
Source: Am J Prev Cardiol. 2026 Apr 19;29:101646. doi: 10.1016/j.ajpc.2026.101646 (PMC13329528; doi:10.1016/j.ajpc.2026.101646)
Supplement: Supplementary file 1 [file mmc1.docx]

## SUPPLEMENTARY TABLES

| **Supplementary Table 1. Baseline Characteristics of the Study Population and Excluded Participants** | | | |
| --- | --- | --- | --- |
|  | **Study population (n=3,477)** | **Excluded population (n=1,584)** | **p-value** |
| Age (years) | 51.0 (45.5–56.7) | 51.0 (39.0–59.0) | <0.001 |
| White | 2058 (59.2%) | 906 (57.2%) | 0.685 |
| College graduate or higher | 1,526 (43.9%) | 748 (54%) | 0.13 |
| Systolic Blood Pressure (mmHg) | 117.5 (107.0–127.5) | 118.5 (107.0–131.0) | 0.447 |
| Body Mass Index (kg/m²) | 26.7 (24.1–29.8) | 26.9 (23.9–30.6) | 0.124 |
| Physically Active | 1,718 (49.4%) | 744 (47%) | 0.12 |
| Hypertension | 833 (23.9%) | 572 (36.1%) | <0.001 |
| Diabetes | 523 (15.0%) | 281 (17.7%) | 0.15 |
| Current Smoking | 565 (16.2%) | 259 (16.4%) | 0.928 |
| Statin Use | 431 (12.4%) | 265 (16.7%) | <0.001 |
| Total Cholesterol (mg/dL) | 199.0 (176.0–223.0) | 188.5 (162.0–219.0) | <0.001 |
| LDL-C (mg/dL) | 118.0 (98.0–140.0) | 109.0 (87.0–134.0) | <0.001 |
| HDL-C (mg/dL) | 51.0 (44.0–60.0) | 50.0 (43.0–59.0) | 0.22 |
| Triglycerides (mg/dL) | 112.0 (81.0–157.0) | 103.0 (72.5–146.0) | <0.001 |
| UACR (mg/g) | 0.5 (0.4–0.8) | 0.5 (0.4–0.9) | 0.41 |
| Hemoglobin A1c (%) | 5.2 (4.9–5.6) | 5.2 (4.9–5.6) | 0.669 |

Baseline demographic and clinical characteristics comparing individuals included in the study population and those excluded from the analytic sample. Variables include cardiovascular risk factors, laboratory measures, and comorbidities. Continuous variables are presented as median (interquartile range) and categorical variables as counts and percentages. Comparisons between groups were performed for all variables using appropriate statistical tests; p-values are reported for each comparison.

**Abbreviations:**
HDL-C = high-density lipoprotein cholesterol; LDL-C = low-density lipoprotein cholesterol; UACR = urinary albumin-to-creatinine ratio; Hemoglobin A1c = glycated hemoglobin.

| **Supplementary Table 2 - Reclassification Table by guideline risk strata** | | | | | |
| --- | --- | --- | --- | --- | --- |
|  | **PCE <5%** | **PCE 5-7.4%** | **PCE 7.5%-19.9%** | **PCE ≥20%** | **TOTAL** |
| **PREVENT <5%** | 2,296(66.0%) | 333(9.5%) | 286(8.2%) | 1(0.0%) | **2,916(83.8%)** |
| **PREVENT 5-7.4%** | 16 (0.5%) | 31 (0.9%) | 240 (6.9%) | 14 (0.4%) | **301 (8.7%)** |
| **PREVENT 7.5%-19.9%** | 3 (0.1%) | 6 (0.2%) | 94 (2.7%) | 148 (4.3%) | **251 (7.2%)** |
| **PREVENT ≥20%** | 0 | 0 | 0 | 9 (0.3%) | **9 (0.3%)** |
| **TOTAL** | **2,315 (66.6%)** | **370 (10.6%)** | **620 (17.8%)** | **172 (4.9%)** | **3,477 (100.0%)** |

This table presents cross-classification of 10-year ASCVD risk categories according to PREVENT and Pooled Cohort Equations (PCE) using guideline-defined strata (<5%, 5.0–7.4%, 7.5–19.9%, ≥20%). Overall agreement, downward and upward reclassification proportions are shown.

Abbreviations: PCE = Pooled Cohort Equations; PREVENT = Predicting Risk of Cardiovascular Disease EVENTs.

| **Supplementary Table 3 - Risk Classification of Participants With CAC ≥100 by PREVENT and PCE** | | | | | |
| --- | --- | --- | --- | --- | --- |
| **Population** | **Total CAC ≥100** | **PREVENT Below Threshold n (%)** | **PCE Below Threshold n (%)** | **PREVENT Above Threshold n (%)** | **PCE Above Threshold n (%)** |
| Overall | 348 | 118 (33.9%) | 127 (36.5%) | 230 (66.1%) | 221 (63.5%) |
| Men | 259 | 75 (29.0%) | 64 (24.7%) | 184 (71.0%) | 195 (75.3%) |
| Women | 89 | 43 (48.3%) | 63 (70.8%) | 46 (51.7%) | 26 (29.2%) |

This table reports the proportion of participants with CAC ≥100 classified below or above the CAC-testing threshold using PCE (7.5%) and PREVENT (3.7% for men; 4.9% for women).

Abbreviations: CAC = coronary artery calcium; CI = confidence interval; PCE = Pooled Cohort Equations; PREVENT = Predicting Risk of Cardiovascular Disease EVENTs.

| **Supplementary Table 4. Risk Classification of Participants With Any CAC (CAC >0) by PREVENT and PCE** | | | | | |
| --- | --- | --- | --- | --- | --- |
| **Population** | **Total CAC >0** | **PREVENT Below threshold n (%)** | **PCE Below threshold n (%)** | **PREVENT Above Threshold n (%)** | **PCE Above Threshold n (%)** |
| Overall | 1,049 | 863 (82.3%) | 563 (53.7%) | 186 (17.7%) | 486 (46.3%) |
| Men | 666 | 300 (45.0%) | 281 (42.2%) | 366 (55.0%) | 385 (57.8%) |
| Women | 383 | 270 (70.5%) | 282 (73.6%) | 113 (29.5%) | 101 (26.4%) |

This table presents the classification performance of standard PCE and PREVENT thresholds for detecting any coronary artery calcium (CAC >0).

Abbreviations: CAC = coronary artery calcium; PCE = Pooled Cohort Equations; PREVENT = Predicting Risk of Cardiovascular Disease EVENTs.

| **Supplementary Table 5. Performance Metrics of PREVENT and PCE for CAC >0 Across Key Threshold** | | | | | | | | | |
| --- | --- | --- | --- | --- | --- | --- | --- | --- | --- |
| **Population** | **Model/Threshold** | **Sens % (95% CI)** | **Spec % (95% CI)** | **PPV %** | **NPV %** | **NNS** | **CAC >0 Detected n (%)** | **Extra Scans per Det** | **Screened %** |
| Both sexes | PCE Borderline (5.0) | 61.3 (58.5–64.0) | 78.6 (77.1–80.0) | 55.3 | 82.5 | 1.8 | 643 (61.3) | 2.3 | 33.4 |
|  | PCE Intermediate (7.5. Ref) | 46.3 (43.5–49.1) | 87.4 (86.3–88.5) | 61.4 | 79.0 | 1.6 | 486 (46.3) | Ref | 22.8 |
|  | PREVENT Youden (2.7) | 67.6 (64.9–70.2) | 75.2 (73.7–76.7) | 54.1 | 84.3 | 1.8 | 709 (67.6) | 2.3 | 37.7 |
|  | PCE Youden (3.1) | 77.7 (75.2–80.1) | 64.6 (63.0–66.2) | 48.7 | 87.0 | 2.1 | 815 (77.7) | 2.7 | 48.1 |
| Men | PREVENT Recommended (3.7. Ref) | 55.0 (51.2–58.7) | 79.0 (76.4–81.4) | 65.7 | 70.5 | 1.5 | 366 (55.0) | Ref | 35.4 |
|  | PREVENT Youden (2.7) | 70.0 (66.5–73.3) | 66.3 (63.3–69.2) | 60.4 | 75.1 | 1.7 | 466 (70.0) | 2.7 | 44.7 |
| Women | PREVENT Recommended (4.9. Ref) | 29.5 (24.9–34.4) | 93.0 (91.7–94.2) | 51.6 | 84.0 | 1.9 | 113 (29.5) | Ref | 11.5 |
|  | PREVENT Youden (2.7) | 63.4 (58.6–68.0) | 80.5 (78.5–82.4) | 45.1 | 89.7 | 2.2 | 243 (63.4) | 2.6 | 19.7 |
|  | PREVENT Youden Female (2.1) | 74.7 (70.3–78.7) | 71.9 (69.6–74.1) | 40.1 | 91.8 | 2.5 | 286 (74.7) | 3.2 | 37.5 |

This table summarizes diagnostic and operational performance metrics for standard and detection-optimized thresholds of PREVENT and PCE for identifying CAC >0. Measures include sensitivity, specificity, PPV, NPV, number needed to screen (NNS), incremental scans per additional CAC >0 detected relative to the reference threshold, and proportion eligible for scanning.

Abbreviations: CAC = coronary artery calcium; CI = confidence interval; NNS = number needed to screen; PCE = Pooled Cohort Equations; PPV = positive predictive value; PREVENT = Predicting Risk of Cardiovascular Disease EVENTs.
